# A human-centered perspective on research challenges for hybrid human artificial intelligence in lifestyle and behavior change support

**DOI:** 10.3389/fdgth.2025.1544185

**Published:** 2025-03-20

**Authors:** Chenxu Hao, Susanne Uusitalo, Caroline Figueroa, Quirine T. S. Smit, Michael Strange, Wen-Tseng Chang, M. I. Ribeiro, Vanita Kouomogne Nana, Myrthe L. Tielman, Maaike H. T. de Boer

**Affiliations:** ^1^ Delft University of Technology, Delft, Netherlands; ^2^ University of Oulu, Oulu, Finland; ^3^ University of California Berkeley, School of Social Welfare, Berkeley, United States; ^4^ TNO, Soesterberg, Netherlands; ^5^ Department of Global Political Studies, Malmö University, Malmö, Sweden; ^6^ Amsterdam University of Applied Sciences, Amsterdam, Netherlands; ^7^ Technical University of Eindhoven, Eindhoven, Netherlands; ^8^ Department of Computer Sciences and Information Systems, Faculty of Sciences and Engineering, University of Limerick, Limerick, Ireland

**Keywords:** human computer interaction, hybrid intelligence, behavior change, healthcare, support system

## Abstract

As intelligent systems become more integrated into people’s daily life, systems designed to facilitate lifestyle and behavior change for health and well-being have also become more common. Previous work has identified challenges in the development and deployment of such AI-based support for diabetes lifestyle management and shown that it is necessary to shift the design process of AI-based support systems towards a human-centered approach that can be addressed by hybrid intelligence (HI). However, this shift also means adopting a user-centric design process, which brings its own challenges in terms of stakeholder involvement, evaluation processes and ethical concerns. In this perspective paper, we aim to more comprehensively identify challenges and future research directions in the development of HI systems for behavior change from four different viewpoints: (1) challenges on an individual level, such as understanding the individual end-user’s context (2) challenges on an evaluation level, such as evaluation pipelines and identifying success criteria and (3) challenges in addressing ethical implications. We show that developing HI systems for behavior change is an interdisciplinary process that requires further collaboration and consideration from various fields.

## 1 Introduction

As technology, particularly intelligent systems, becomes more integrated into people’s daily life, systems designed to facilitate lifestyle or behavior change for health and well-being have also become more common [e.g., personal informatics systems for fitness and health (1), virtual coach for initial goal setting in behavior change (2)]. However, there is still a long process going from research that develops such support systems to deploying such systems in people’s everyday life (3).

Particularly, the development and deployment of AI-based support systems in healthcare and well-being encounter challenges from various sources. For example, developing support systems for diabetes lifestyle management faces challenges in continuous involvement of diverse stakeholder, long-term patient engagement, management of evolving domain knowledge, and accounting for interdependence (3).

This calls for a shift of the design towards a human-centered and interdisciplinary approach, which can be addressed by the role of Hybrid Intelligence [HI, (4)]. In a HI-system, human capabilities are augmented by their complementary AI capabilities, thus achieving improved results overall (4, 5). HI provides a human-centered framework in developing methods for adaptive stakeholder involvement, inclusive interaction capabilities and facilitating collaborative support for knowledge management, which has great potential for health behavior support systems (3). However, developing HI systems for such systems also calls for new development processes, which put the users center stage, to ensure that their individual needs are taken into account, that they can trust the outcomes of user-centric evaluations, and that the entire process happens in an ethical and responsible way.

In this paper, we provide our interdisciplinary perspective on the challenges in the *development process* of HI systems for behavior change and lifestyle support from three different levels: (1) challenges on an individual level, such as challenges to the stakeholders involved in the research, and understanding the individual end-users, (2) challenges on an evaluation level, such as how to properly evaluate such systems and identify success criteria, and (3) challenges in addressing ethical implications during development.

## 2 Background

### 2.1 Hybrid intelligence

Hybrid Intelligence (HI) is a human-centered approach that combines human and artificial intelligence to augment human intelligence (4–6). As an emerging research & development field, few comprehensive HI applications have been implemented (7). However, such joint human-AI systems provide us with potential to realize better performance for the stakeholders at the levels of individuals, group, and society (8).

HI is slightly different, though related to what is often called Hybrid AI (9–12), which combines data-driven and knowledge-driven methods in decision-making processes. This approach leverages both the advantages of data-driven discoveries and knowledge of experts. HI systems can often be hybrid in this way as well to leverages these advantages, — a capability that is crucial for the development of intelligent systems in the healthcare domain (8, 13, 14).

### 2.2 Related works

Existing works that propose or implement HI support systems for lifestyle management often emphasize three aspects: (1) the system’s ability to provide sustained support to users, (2) the system’s ability to provide personalized support, and (3) the system’s ability to leverage the expert’s opinions and support the expert’s involvement.

For example, de Boer et al. (8) provide a vision on HI for diabetes lifestyle management that includes maintaining and updating holistic patient profiles through long-term personalized interactions, consulting with healthcare professionals (HCPs), and enabling shared decision-making processes on the goals for the patients between the patients and HCPs. Chen et al. (15) develop a HI support system for lifestyle change that focuses on personalization, combining components for dialogue generation, information extraction and reasoning in order to realize continuous, personalized interactions. de Greeff et al. (16) propose the FATE (FAir, Transparent, Explainable) system that uses knowledge from fair AI, hybrid AI, explainable AI, user interaction and secure learning. The prototype provides personalized explanations for users, using knowledge engineering and federates learning in a setting for diabetes type 2.

While current work already focuses on enabling intelligent systems to provide continuous, adaptive support for individual users, combining user data and existing knowledge from the relevant support domains, the development of such HI systems still face additional challenges from various aspects. In particular, this work focuses on the *development process* of HI systems for behavior support in lifestyle settings. Within this process, rather than focusing on the technical implementation details, we identify three main *user-centric* levels. The first is related to individual stakeholders in this development, identifying who these are, and what the challenges are for them. The second is related to the evaluation processes, which users need to be able to depend on for safe health systems. The final is the ethical and responsible development dimensions, which should be considered throughout the process.

## 3 Research challenges for HI in lifestyle and behavior change support

### 3.1 Research challenges on the individual-level

#### 3.1.1 Identifying individual stakeholder roles

We take the perspective of de Greeff et al. (16), de Boer et al. (8, 17) to identify three main stakeholder roles: researcher, consultant and subject.

The (AI) researcher is a data scientist or AI developer, interested to learn and object knowledge from data. An example of a challenge for the researcher is that the conditions and user requirements are not easily implemented in a system, and proper evaluation is difficult, especially in a healthcare setting.

The consultant is a domain expert who holds expert knowledge in a particular field, such as medicine. The goal of the consultant is to advice or intervene with the data of the subject, such as activities or lifestyle. The consultant uses the system to obtain contextual information and question the subject for further information. In lifestyle advice, healthcare professionals face many challenges, such as a shortage of time, difficulty understanding new systems and their trustworthiness,

The subject is a “naive” user, subjected to the output of the system or an interest in the output. This user is often not schooled in AI or the domain. In the healthcare domain, this is typically the patient. All patients have a different interest, so personalization of the system or at least the user interface and explanation of the output is necessary.

#### 3.1.2 Individual challenges

As the development process of HI support systems typically involves the stakeholders identified above and each role holds different background knowledge, needs, and expectations to the system, it is crucial to understand the context of each stakeholder when developing and deploying a HI support system. Here, we discuss some contextual challenges in creating an HI system, particularly in the context of system development process (researcher and consultant roles) and the context of end users (subject role).

Developing HI support systems for lifestyle and healthcare is an interdisciplinary field, and researchers and consultants (e.g., HCPs) usually come from various research contexts. These stakeholders often have different expectations for what a HI system could contribute (18), leading to different contextual challenges. For researchers, the main challenges lie in understanding and balancing the needs of both consultants and end users. For example, when developing an AI-based healthcare project for vulnerable groups, such as people with dementia, it is important for researchers and developers from technical backgrounds to understand the subjects, i.e., end users, create and follow a simulation protocol before conducting a field experiment, and test healthcare-related products in a simulated environment before involving vulnerable users (19).

Consultants play an important role in helping researchers tailor the products to meet the needs of end-users. They face the challenge of understanding the capabilities and limitations of the system. By understanding the system, they could bridge the gap between the technical solution and the user requirements.

End-users’ challenges revolve less about challenges to them in developing systems, but rather about HI systems understanding them. Recognizing the end-users’ role in HI design could improve the system’s effectiveness by allowing for personalization based on a deeper understanding of their context. This context includes community, inter-personal, and intra-personal variations, all essential for designing health behavior change support tools.

Community variations address the needs of different groups. Inter- and intra-personal variations focus on the unique needs of individuals within a community and over time. In healthcare scenarios, users’ physiological conditions, reactions, or perceptions can significantly differ from healthy individuals due to factors such as cognitive impairments or specific medical conditions (e.g., diabetes or visual impairments). One of the key challenges in developing feasible HI applications is ensuring that the system can recognize and adapt to the unique needs and lifestyle of each user. For example, if the system needs to explain its decisions for users with a cognitive impairment, it should adjust these to be understandable to the individual (20). Current techniques cannot continuously collect personal and contextual data in real-time to deliver adaptive interventions effectively (21). An ideal HI system could collaborate with the user, evolving and adapting to their changing needs and desires over different life stages (22). This interactive process is bidirectional: While users gradually become familiar with the system and learn how to use it, the system simultaneously learns about the unique context, preferences, and behaviors of the user, motivating and empowering the users in their daily life. However, how to properly develop this process remains an open challenge.

Another challenge is that users’ expectations of AI support systems often conflict with their privacy needs. AI systems rely on monitoring data to support behavior change through recommendations. When users prioritize privacy, AI systems may be limited in their support capabilities. Ideally, we would develop HI systems where users can monitor their own data in a trustworthy manner and optimize their behavior recommendations.

### 3.2 Evaluation challenges

#### 3.2.1 Infrastructure and system evaluation

To develop an ethical HI system for human interaction, the infrastructure must be stable and transparent. Identifying vulnerabilities and balancing benefits and harms, particularly for end-users and other stakeholders, is crucial. In healthcare, handling sensitive data requires adherence to regulatory frameworks. While the EU’s regulatory sandbox process is ongoing, its role in healthcare remains unclear (23). Nevertheless, a holistic assessment beyond data protection impact is essential for such complex settings such as healthcare.

Technical system evaluation can be performed in a few stages: in silico (simulation), in vitro (in research laboratory, test run), and in vivo (in the final destination, e.g., patients home, hospital etc.). The in vivo testing, in particular, requires ethical scrutiny.

While such evaluation process seem problematic due to a great amount of resources it requires in terms of many stages, time and precautionary measures (e.g., addressing privacy issues), this kind of evaluation process can be necessary in many human-centered research domains. For example, in drug development, before the first-in-human studies in clinical drug trials, there is already a long process with different stages.

Analogous to drug development, securing healthcare approval for HI systems involves lengthy and costly certification by regulatory agencies (e.g., EMA in Europe) to assess safety and efficacy. This process limits engagement to certain researchers and often lags behind the rapid evolution of the field, leaving certified technologies potentially outdated. This discourages innovation and can make the effort seem futile.

Another challenge is collecting enough data for reliable results, which requires a great amount of participants. Similar to the case with drug trials, especially in latter stages where it would be essential to have big populations in order to find out long-term outcomes and risks.

Similar to how culture and ethnicity influence the effectiveness of medicine, HI systems designed for diverse cultures may face challenges with value alignment of the product and users. Including individuals from varied groups should be prioritized rather than minimizing differences in the research population. While homogeneous populations may yield clearer research results, they are less effective for real-world implementation, where populations are far more diverse.

#### 3.2.2 Formulating success over process changes

Besides infrastructure, another challenge in developing and evaluating HI systems for behavioral change support is the difficulty in formulating the success of such a HI system. For example, when evaluating a system for behavioral change in diabetes patients for health improvement, there are several factors at play. Firstly, there is a complex link between behavior change and health outcomes (24). Secondly, these types of systems are a type of complex interventions, meaning that there are many (social) factors that can affect the health outcome (25, 26).

The complexity of the link between behavior and the various health outcomes is a common challenge in the field of behavior change. The evidence for the link is stronger in some behaviors than in others. However, for each health behavior there is a complex relationship to health outcomes (24). Klasnja et al. (27) argue that due to this, measuring the effectiveness of new technologies should not be limited to whether the intended behavior or health change happened.

HI systems for healthy behavior change are an example of a complex intervention. These are health service interventions that are not drugs or surgical procedures, but are made up of various interconnecting parts (25, 26). Because of their comprehensive nature and the interconnect with social context, complex interventions pose challenges. It is important to evaluate the process of a behavior change support system (28). The outcome can be influenced by the extent to which the intervention is applied correctly, how the patient engages with the intervention and the social setting of the intervention.

Thus, evaluating HI systems for behavioral change is a complex issue with many facets which requires an interdisciplinary approach.

### 3.3 Ethical implications in HI system development

#### 3.3.1 The trade-off between focusing on individual improvement and focusing on public health improvement

When discussing HI-systems that work in the context of healthcare, it is important to keep the two levels of healthcare in mind: the individuals’ right to healthcare when facing (severe enough) health problems and public health—that is, taking all individuals and their health to be the issue. With new innovations, the aim is to achieve goals that benefit the users and their intended goals (e.g., when HI-system concerns the consultants, their aim typically is to achieve health benefit for a patient or a population). This sometimes may be in conflict with public health. According to the United Nations’s declaration, everyone has a human right to life and this means for instance in healthcare that everyone should be treated when facing a life-threatening problems.

Sometimes the individual and the public health level are in conflict because not all cases of addressing individual problems promote public health. Very expensive interventions that produce only sight improvement in individuals may be of great significance to those very individuals but at the same time, the alternative costs of those measures may be away from some other service and thus bring about health costs to others by limiting the service. HI-systems should be aware of these contexts and explore their potential in both domains. This basically means that when assessing and evaluating the benefits for the intended stakeholders, it should also be considered what are the likely alternative costs and to whom do they fall. Making these analyses facilitates decision-making that can be fair and inclusive on population level as well as the individual one.

#### 3.3.2 The risk of increasing healthcare inequities

AI in healthcare is often seen as a way to improve access for those facing systemic barriers, such as low-income populations or areas with doctor shortages. However, digital health interventions have a record of exclusive rather than inclusive design and research practices. For example, the majority of digital health innovations have been tested and used by individuals who are higher-income, white, female and higher educated.

This lack of an equity lens in design has led to significant disparities in digital health access and use. For example, in Europe, digital health tools are more commonly used by younger, urban, higher socioeconomic groups, while access is lower for people with disabilities or language barriers.

To address this, HI systems design should prioritize a focus on co-design and participatory design, where users are involved in all design stages of the system. For instance, a diabetes decision-making tool designed without considering disadvantaged users might fail to support those facing financial struggles or disabilities, despite being effective for more privileged groups.

#### 3.3.3 Addressing diversity, equity, inclusion

Acknowledging individuals’ diversity and designing interventions that respond in an equitable fashion to those needs and challenges is the central challenge for creating successful healthcare systems. It is in that context that diverse and equitable inclusion (DEI) must be considered when designing and implementing HI technologies in healthcare. To the extent that AI is a connective technology able to utilize and see patterns within big data sets, it has the capacity to support diverse inclusion on the basis of “seeing more” as well as “needing more” data. Yet, that is not the same as equity and, certainly, we see machine learning systems replicating inequities present within the data taken from an inequitable society. Calls for “human-in-the-loop” quality checks to identify mis-learnt biases speak to the hybrid aspect of HI but it is far from clear that humans can effectively perform that function given the scale of data utilized in healthcare and on which machine learning systems are trained. Furthermore, if we are to show fidelity to the principle of equity, it requires building diversity into the entire development ecosystem such that there can be HI. Without equity, HI becomes limited to only a few—likely already privileged—humans unable to understand, or unaware of, the needs and obstacles faced by other individuals.

## 4 Conclusion

In this perspective article, we have identified several challenges in developing HI support systems for lifestyle and behavior change in healthcare setting. In addition to what we have highlighted, there are still additional challenges in technical development of systems, embedding of systems in societal and healthcare settings while balancing stakeholders, and so on. We argue that HI system development in healthcare is an interdisciplinary process with various future directions related to tackling these challenges, including conducting detailed case studies and pilot applications. Such development requires collaborations and discussions from various fields.

## Data Availability

The original contributions presented in the study are included in the article/Supplementary Material, further inquiries can be directed to the corresponding author.
